# Immunoproteasome Inhibition Reduces the T Helper 2 Response in Mouse Models of Allergic Airway Inflammation

**DOI:** 10.3389/fimmu.2022.870720

**Published:** 2022-05-30

**Authors:** Franziska Oliveri, Michael Basler, Tata Nageswara Rao, Hans Joerg Fehling, Marcus Groettrup

**Affiliations:** ^1^ Division of Immunology, Department of Biology, University of Konstanz, Konstanz, Germany; ^2^ Biotechnology Institute Thurgau at the University of Konstanz, Kreuzlingen, Switzerland; ^3^ Institute of Immunology, University Hospital, Ulm, Germany

**Keywords:** allergic airway inflammation, eosinophilia, GATA-3 reporter mice, immunoproteasome inhibition, Th2 cells

## Abstract

**Background:**

Allergic asthma is a chronic disease and medical treatment often fails to fully control the disease in the long term, leading to a great need for new therapeutic approaches. Immunoproteasome inhibition impairs T helper cell function and is effective in many (auto-) inflammatory settings but its effect on allergic airway inflammation is unknown.

**Methods:**

Immunoproteasome expression was analyzed in *in vitro* polarized T helper cell subsets. To study Th2 cells *in vivo* acute allergic airway inflammation was induced in GATIR (GATA-3-vYFP reporter) mice using ovalbumin and house dust mite extract. Mice were treated with the immunoproteasome inhibitor ONX 0914 or vehicle during the challenge phase and the induction of airway inflammation was analyzed.

**Results:**

*In vitro* polarized T helper cell subsets (Th1, Th2, Th17, and Treg) express high levels of immunoproteasome subunits. GATIR mice proved to be a useful tool for identification of Th2 cells. Immunoproteasome inhibition reduced the Th2 response in both airway inflammation models. Furthermore, T cell activation and antigen-specific cytokine secretion was impaired and a reduced infiltration of eosinophils and professional antigen-presenting cells into the lung and the bronchoalveolar space was observed in the ovalbumin model.

**Conclusion:**

These results show the importance of the immunoproteasome in Th2 cells and airway inflammation. Our data provides first insight into the potential of using immunoproteasome inhibition to target the aberrant Th2 response, e.g. in allergic airway inflammation.

## Introduction

Asthma is a chronic disease affecting more than 300 million people worldwide which is characterized by breathing difficulties due to airway inflammation (AI), mucus hypersecretion and airway wall thickening (1). Allergic asthma, the most common form, is induced by inhalation of environmental allergens which trigger an excessive immune response resulting in high levels of inflammatory cytokines like interleukin- (IL-) 4, IL-13 and IL-5, eosinophilic infiltration, mast cell activation and increased immunoglobulin E (IgE) production by B cells (2). Antigen-specific T helper (Th) 2 cells are central mediators of this allergic reaction. Therapeutic approaches aim to reduce Th2 responses, e.g. inhaled corticosteroids are most commonly used for long-term medication which reduces many aspects of the Th2 response (3). Even though most patients benefit from corticosteroids, a large proportion of patients develops corticosteroid-resistant asthma leading to the great need of novel therapeutics (4). New approaches target mediators like IL-4 and IL-5 (5) which proved most effective when given in combination, highlighting the relevance of the Th2 cytokines and the necessity to target several pathways.

Besides its role in antigen presentation (6), many studies have shown the importance of the immunoproteasome in activation and differentiation of T cells, cytokine secretion and development of autoimmune disorders (7–15), making it an interesting therapeutic target for T helper cell mediated diseases. The immunoproteasome is a form of the 26S proteasome in which the standard catalytically active β-subunits (β1c, β2c and β5c) are replaced by low molecular mass polypeptide (LMP)2 (β1i), multicatalytic endopeptidase complex-like (MECL)-1 (β2i) and LMP7 (β5i). These subunits are constitutively expressed in hematopoietic cells and can be induced by interferon-γ (IFN-γ) in other tissues (16–19). Irreversible inhibition of the immunoproteasome subunits LMP2/LMP7 by ONX 0914 has shown great effects in ameliorating disease symptoms in various mouse models of inflammatory diseases (9–12, 20, 21). These studies could show that immunoproteasome inhibition reduces CD4^+^ IFN-γ^+^ Th1 and CD4^+^ IL-17^+^ Th17 cells in experimental autoimmune encephalomyelitis (12) and dextran sodium sulfate-induced colitis (9). In line, immunoproteasome inhibition reduced cytokine expression and secretion associated with Th1 and Th17 cells in various models and blocks Th17 differentiation *in vitro* (9–12, 22–24). In contrast, little is known about the role of the immunoproteasome in the development of allergic Th2 mediated inflammation. Volkov *et al.* showed that LMP7-deficient mice develop a less pronounced Th2 response compared to wildtype mice in ovalbumin (OVA)- but not in house dust mice (HDM)- induced AI (25). To our knowledge, there are no studies investigating the effect of immunoproteasome inhibition on Th2 differentiation in allergic settings.

This study aims at assessing the role of the immunoproteasome in Th2 cells and the therapeutic potential of immunoproteasome inhibition on Th2 cells in allergic AI. We found that Th2 cells express high levels of the immunoproteasome when polarized *in vitro*. To investigate the therapeutic effect *in vivo*, we used the novel GATA-3-reporter strain GATIR (26) to easily identify Th2 cells. We could show a reduction of the Th2 response upon ONX 0914 treatment in both, OVA- and HDM-induced AI models.

## Material and Methods

### Mice

GATIR (Gata3^tm1.1Hjf^ (26),) mice were kindly provided by Hans Joerg Fehling (Institute of Immunology, University Clinics Ulm, Ulm, Germany). IL-17A-GFP (C57BL/6-*Il17a^tm1Bcgen^*/J; stock #018472) mice were purchased from The Jackson Laboratories (27). C57BL/6 mice were originally purchased from Charles River Laboratories. LMP7- (Psmb8^tm1Hjf^ (28),) and MECL-1- (Psmb10^tm1Mgro^ (29),) gene targeted mice were kindly provided by Dr. J. J. Monaco (Department of Molecular Genetics, Cincinnati Medical Center, Cincinnati, OH) and LMP7-MECL-1-double-deficient mice were obtained by crossing. Male and female mice were used at 8-14 weeks of age.

### *In Vitro* T Cell Polarization

T cells were isolated using the naïve CD4^+^ T cell isolation kit (Miltenyi Biotech) according to the manufacturer’s instructions from spleens of naïve IL-17A-GFP and GATIR mice. Polarization was induced by addition of cytokine/antibody cocktails using the Cytobox kits for Th1, Th2 and Th17 (Miltenyi Biotech) according to the manufacturer’s instructions with the exception of using plate bound anti-CD3/anti-CD28 (antibody list in **Supplementary Table S1**) and RPMI medium containing 10% FCS, 1x penicillin/streptomycin and 50 µM β-mercaptoethanol. Tregs were activated using plate bound anti-CD3/anti-CD28 and polarized with 15 ng/ml TGF-β1, 10 ng/ml IL-2, 5 µg/ml anti-IFN-γ and 5 µg/ml anti-IL-4. On day 7, cells were harvested and analyzed by immunoblotting and flow cytometry as described below.

### Immunoblotting

*In vitro* polarization samples or dissociated mouse tissue samples were lysed in RIPA buffer (50 mM Tris-buffered HCl, pH 7.5 containing 150 mM NaCl, 1% NP-40, 0.5% SDS) for 20 min on ice and protein content was determined by BCA assay (ThermoFisher). 15-20 µg protein lysate was separated by SDS-PAGE and transferred onto a nitrocellulose membrane. Expression of the immunoproteasome subunits was analyzed by incubation with antibodies directed against LMP7, LMP2 and MECL-1 as well as the constitutive subunits β1c, β2c and β5c (see **Supplementary Table S1**). Anti-γ-tubulin and anti-GAPDH served as loading controls. IRDye800CW goat anti-rabbit or anti-mouse and IRDye680RD goat anti-mouse or anti-rabbit (LI-COR) were used as secondary antibodies. Signals were analyzed with the LI-COR Odyssey Imager and the Image Studio Lite Version 5.2.

### Induction of Airway Inflammation With Ovalbumin

AI was induced by intraperitoneal (i.p.) sensitization followed by aerosol challenge using chicken egg ovalbumin (OVA) as described before (30). In short, mice received two i.p. injections of 50 µg ovalbumin (OVA grade V; Merck) dissolved in phosphate buffered saline (PBS) and mixed 1:1 with Imject Alum (ThermoFisher) on day 0 and 7. On day 14, 15 and 16 mice were challenged with nebulized 1% OVA solution (in ddH_2_O) for 20 min using the Aerosol Jet-Nebulizer (Hugo Sachs-Elektronik, Harvard Apparatus GmbH). PBS control mice received two i.p. injections of PBS/Alum on day 0 and 7 and were challenge with PBS only on days 14, 15 and 16. Mice were sacrificed and analyzed on day 17. The “challenge only” control group was not immunized with OVA/Alum, but challenged three times with 1% OVA aerosol and analyzed one day after the last challenge.

### House Dust Mite Induced Airway Inflammation

For induction of house dust mite (HDM) AI, we dissolved D. pteronyssinus extract (Citeq Biologicals) in PBS and applied 50 µg intranasally on day 0, 7, 14 and 21 to anesthetized mice as described before (25). Mice were sacrificed on day 23 to analyze airway inflammation. In one experiment, mice received only three applications of HDM on day 0, 7 and 14 and were analyzed on day 21 (group “d21”).

### Proteasome Inhibition in Mice

ONX 0914 (10) (Kezar Life Sciences) was formulated in a solution of 10% sulfobutylether-β-cyclodextrin and 10 mM sodium citrate (pH 6; vehicle). 10 mg/kg was administered subcutaneously. In OVA-AI experiments, mice received injections on day 12 and always one hour before each aerosol challenge (day 14, 15, 16). For HDM experiments, mice received a single dose on day 21 1 h before the intranasal challenge, except for group “d21” which did not receive any treatment. Control mice received the same volume of vehicle.

### Bronchoalveolar Lavage

Mice were sacrificed, the trachea exposed and a small incision was made to insert a blunted cannula. The lung was flushed three times using 0.8 ml PBS each time. The bronchoalveolar lavage fluid (BALF) containing the infiltrating cells was collected, viable cells were counted using the Cellometer Auto 2000 Cell Viability Counter (Nexcelom Bioscience) and analyzed by flow cytometry.

### Serum Collection and Determination of Antibody Concentration

Blood was collected from sacrificed mice by cardiac puncture and transferred into serum collection tubes (Sarstedt). Samples were centrifuged to separate the serum which was frozen at -80°C till analysis. Antibody concentrations were measured using the Mouse Anti-OVA IgG1 Antibody ELISA Kit (Chondrex) and the LEGEND MAX™ Mouse OVA Specific IgE ELISA Kit (Biolegend).

### Organ Preparation and Flow Cytometry

Spleens were collected and single cell suspensions were prepared using 70 µm nylon mesh. Lungs were dissected into single lobes and briefly washed with PBS. Single cell suspensions were obtained using the Lung Dissociation Kit and the gentleMACS Octo Dissociator according to the manufacturer’s instructions (Miltenyi Biotec). After erythrocyte lysis, F_c_-receptor blocking was performed using the 2.4G2 antibody for 20 min at 4°C. Cells were washed once and subsequently stained with different antibody cocktails (list of antibodies in **Supplementary Table S1**) for 30 min at 4°C followed by three washing steps. Sytox Blue/Red (ThermoFisher) staining was included for live/dead cell discrimination. For intracellular stainings, surface staining was performed first along with fixable viability stain 780 (BD Pharmingen) according to the manufacturer’s instructions, followed by fixation with 4% paraformaldehyde for 5 min at 4°C. Samples were subsequently permeabilized with PBS-based buffer containing 0.1% saponin, 2% FCS, 2 mM EDTA, 2 mM NaN_3_. Samples were stained intracellularly with antibodies directed against Ki-67 and FoxP3 overnight at 4°C. After washing, all samples were measured on LSRFortessa and FACSLyric flow cytometers (both BD Biosciences). Flow cytometry data was analyzed with FlowJo v10 (BD Biosciences).

### OVA Restimulation

Spleen and mediastinal lymph nodes of vehicle or ONX 0914 treated OVA-AI mice were used for restimulation with OVA to evaluate the antigen-specific immune response. Organs were dissociated using 70 µm and 150 µm nylon mesh and erythrocyte lysis was performed for spleen samples. 5x10^6^ cells were seeded in a 24-well plate and incubated for 4 days in the presence of 200 µg/ml OVA and 10 ng/ml IL-2. Medium was replaced with fresh medium after 2 days. Cells were harvested, restimulated with PMA/ionomycin/BFA and intracellular cytokine staining was performed.

### PMA/Ionomycin Restimulation and Intracellular Cytokine Staining

Cytokine production was analyzed in samples from *in vitro* polarization, OVA restimulation and lungs from airway-inflammation experiments. Single cell suspensions were seeded in a 96-well plate and restimulated with a mix containing 25 ng/ml phorbol-12-myristat-13-acetat (PMA), 500 ng/ml ionomycin and 10 µg/ml brefeldin A (BFA; all Merck) for 5 hours at 37°C, 5% CO_2_. Afterwards, cells were harvested and surface staining was performed with anti-CD4 and fixable viability stain 780 followed by fixation as described above. Intracellular staining was performed with antibodies directed against IL-4, IL-13 and IFN-γ. Samples were measured on a LSRFortessa flow cytometer (BD Biosciences). Flow cytometry data was analyzed with FlowJo v10 (BD Biosciences).

### Histology

Lungs were dissected into individual lobes and immediately fixed in 10% formalin. After fixation in 10% formalin, samples were embedded in paraffin and sections of 4 µm were used for histological analyses. Samples were stained with hematoxylin and eosin (H&E; Carl Roth) to evaluate the perivascular and peribronchial infiltration as described before (31). Scores were assigned in a blinded manner according to the following criteria: 0 – normal, 1 – few cells, 2 – ring of one cell layer of inflammatory cells, 3 – ring of inflammatory cells 2-4 cells deep, 4 – ring of inflammatory cells of more than 4 cells. Eosinophilic infiltration was analyzed by staining with 0.5% CongoRed (Merck) and counterstaining with hematoxylin. Quantification of CongoRed stain positive area was performed using color deconvolution with ImageJ software. Periodic acid-Schiff (PAS) staining with hematoxylin counterstaining was performed according to the manufacturer’s instructions (Carl Roth) and scored depending on the quantity of PAS+ goblet cells: 0: < 5% goblet cells; 1: 5 to 25%; 2: 25 to 50%; 3: 50 to 75%; 4: > 75%. The sum of airway scores from each lung was divided by the number of airways examined, minimum 5 per mouse, and expressed as PAS score in arbitrary units (U). All images were captured using the Zeiss AxioPlan2 microscope.

### Statistics

Data was analyzed using GraphPad Prism Version 9 and depicted as mean ± standard deviation (SD). Individual data points represent different mice. Normality tests (Kolmogorov-Smirnov and Shapiro-Wilk) were performed to check for Gaussian distribution. In case of normal distribution, Student’s t-test, one- or two-way-ANOVA was performed to determine statistical significance of differences. Holm-Sidak test for multiple comparison was used to compare different groups. Non-normally distributed data was analyzed by Kuskal-Wallis-test. Statistical significance was achieved when p < 0.05; * p < 0.05, ** p < 0.01, *** p < 0.001, and **** p < 0.0001.

## Results

### The Immunoproteasome Is Strongly Expressed in Different T Helper Cell Subsets

**Figure 1:**
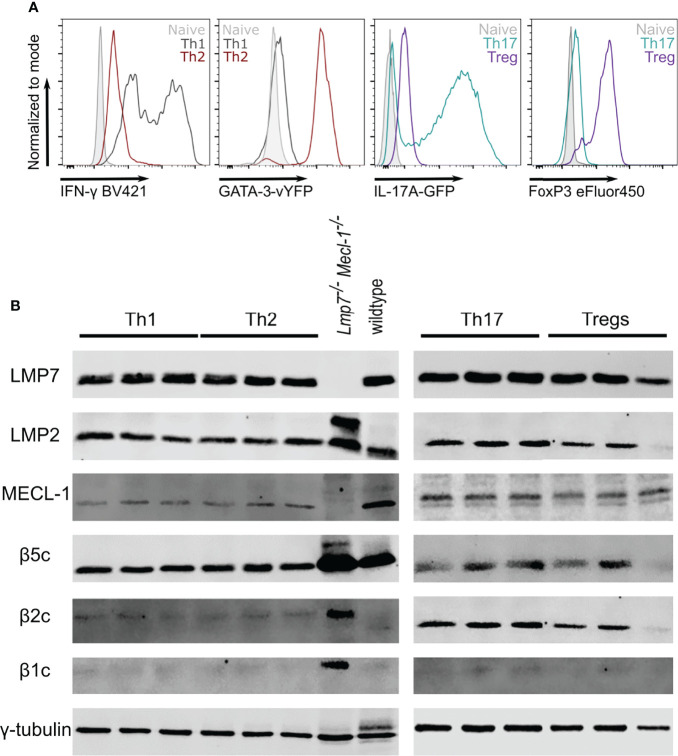
The immunoproteasome is strongly expressed in T helper cell subsets. Naïve CD4^+^ T cells were magnetically sorted from spleens of GATIR and IL-17A-GFP mice and cultured with polarizing cytokine/antibody cocktails for 7 days. **(A)** Flow cytometric analysis of polarization on day 7. Intracellular staining for IFN-γ and FoxP3 was performed for Th1 and Tregs, respectively. GATA-3 and IL-17A expression was analyzed by measuring the respective reporter fluorochrome vYFP or GFP. Histogram overlays show fluorescence levels in the different Th populations with naïve CD4^+^ T cells on day 0 as controls (grey). For each parameter, one other population is shown to demonstrate the specificity of the signal (Th1 – black, Th2 – red, Th17 – petrol, Treg – purple). **(B)** Polarized cells were harvested and lysed on day 7. Lysates were analyzed for LMP7, LMP2, MECL-1, β5c, β2c and β1c by immunoblotting. γ-tubulin was used as loading control. Lanes represent extracts from three different mice. Whole spleen lysates of wildtype (WT) and LMP7-MECL-1-double-deficient mice were used as controls. Note the additional band of LMP2 in the LMP7-MECL-1-double-deficient mice which likely represents the accumulated precursor that is not incorporated into the mature proteasome in the absence of LMP7 and MECL-1.

In order to investigate Th2 cells as potential targets for immunoproteasome inhibition, we used *in vitro* polarized T helper cells to analyze the immunoproteasome content in different subsets. Successful polarization was confirmed by flow cytometric analysis of IFN-γ (Th1), GATA-3 (Th2), IL-17A (Th17) or FoxP3 (Treg) expression (**Figure 1A**). Like unpolarized CD4^+^ T cells (8), we found strong expression of the immunoproteasome subunits LMP7, LMP2 and MECL-1 in all analyzed subsets (**Figure 1B**). Interestingly, the expression of the constitutive subunits β5c, β2c and β1c was only weakly detectable with the exception of β5c in Th1 and Th2 and β2c in Th17 and Tregs.

### Th2 Cells can be Easily Identified in GATIR Reporter Mice

**Figure 2:**
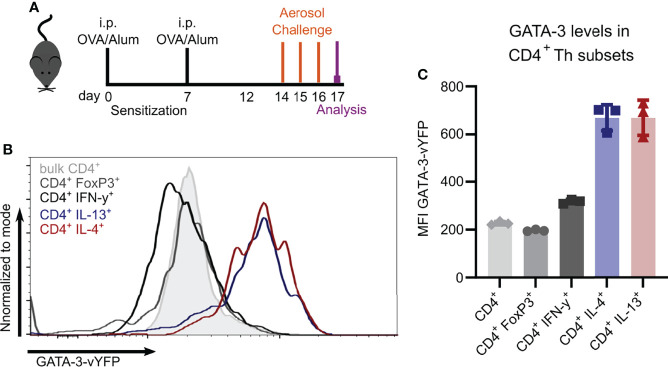
GATA-3-vYFP expression signal correlates with Th2 cytokine expression. GATIR mice were sensitized with OVA/Alum by two intraperitoneal (i.p.) injections on day 0 and 7. On day 14, 15 and 16 they were challenged with aerosolized OVA for 20 min and analyzed on day 17. **(A)** Experimental setup. **(B)** GATA-3-vYFP expression levels in indicated CD4^+^ T cell subsets in the lung compared to bulk CD4^+^ T cells (grey filling) and **(C)** quantification of the mean fluorescence intensity (MFI). Data is shown as mean ± SD, n=3; statistical differences are not indicated for better visibility.

To study Th2 cells *in vivo*, we evaluated the usage of the recently described GATIR-reporter mice (26) since conventional identification of Th2 cells requires intracellular staining, thus limiting the analysis of viable cells. GATIR mice harbor an IRES–vYFP expression cassette in the 3’-untranslated region of the endogenous GATA-3 locus (26). GATA-3 is the transcription factor required for Th2 differentiation which is strongly upregulated in Th2 cells (26, 32–34). To identify Th2 cells *in vivo*, we induced OVA-AI (**Figure 2A**) and analyzed GATA-3-vYFP expression in different Th subsets in the lung. In contrast to bulk CD4^+^ T cells and FoxP3^+^ Tregs, the GATA-3-vYFP level was increased approximately 3-fold in IL-4 and IL-13 expressing CD4^+^ Th2 cells (**Figures 2B, C** **)**. IFN-γ^+^ CD4^+^ Th1 cells exhibited slightly higher GATA-3-vYFP signals than bulk CD4^+^ and Tregs but still less than half of the Th2 cells. These results indicate that vYFP positive cells can be identified as GATA-3 expressing Th2 cells.

### Immunoproteasome Inhibition During the Challenge Phase Reduces Th2 Cells in Ovalbumin-Induced Airway Inflammation

**Figure 3:**
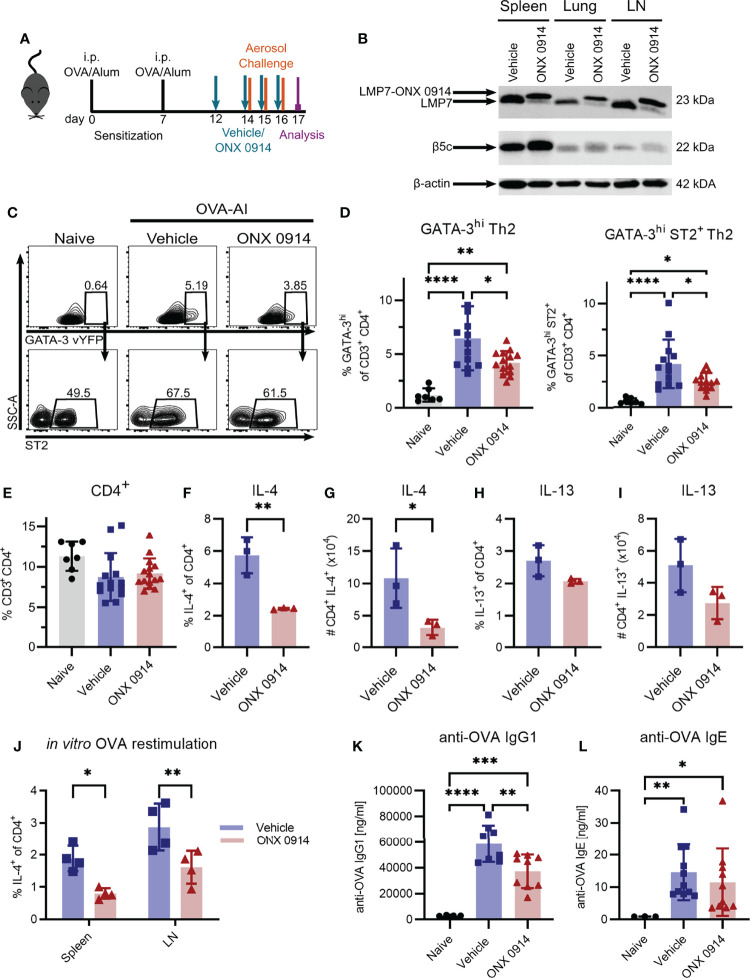
Immunoproteasome inhibition reduces the Th2 response in OVA-induced airway inflammation. GATIR mice were sensitized with OVA/Alum by two intraperitoneal (i.p.) injections on day 0 and 7. On day 14, 15 and 16 they were challenged with aerosolized OVA for 20 min. Mice received subcutaneous injections of 10 mg/kg ONX 0914 or vehicle on day 12, 14, 15 and 16 and were analyzed on day 17. Naïve mice served as controls. **(A)** Experimental setup. **(B)** Lysates of spleen, lung and lymph nodes (LN) were analyzed by immunoblotting against the indicated proteins. The shift of electrophoretic mobility of LMP7 results from covalent modification with ONX 0914. β-actin served as a loading control. **(C)** Gating strategy for the identification of Th2 cells in the lung as GATA-3^hi^ and GATA-3^hi^ co-expressing ST2 after doublet and dead cell exclusion and pre-gating on CD3^+^ CD4^+^. **(D)** Quantification of the frequencies. **(E)** Frequency of total CD3^+^ CD4^+^ T cells in the lung. Frequency **(F, H)** and absolute count **(G, I)** of IL-4 and IL-13 expressing CD4^+^ T cells in the lung. **(J)** Cells from spleen and lymph nodes were restimulated with ovalbumin for 4 days *in vitro* to measure the frequency of antigen-specific IL-4^+^ CD4^+^ cells. Concentrations of anti-OVA IgG1 **(K)** and IgE **(L)** in the serum. Data is shown as mean ± SD and statistical significance was determined depending on the data structure. **(D, E)** Pooled data from 5 independent experiments (naïve: n=7; vehicle/ONX 0914 n=13-14) was analyzed by one-way ANOVA with Holm-Sidak test for multiple comparison. **(F-I)** Representative result of two independent experiments, n=3, data was analyzed by two-tailed t test. **(J)** Representative result of two independent experiments, (n=4) data was analyzed by two-way ANOVA with Holm-Sidak test for multiple comparison. **(K)** Pooled data of three independent experiments (naïve: n=4; vehicle: n=8; ONX 0914: n=9) was analyzed by one-way ANOVA and Holm-Sidak test. **(L)** Pooled data of three independent experiments (naïve: n=4; vehicle: n=11; ONX 0914: n=10) was analyzed by Kruskal-Wallis test (not normally distributed). *p <0.05, **p < 0.01, ***p < 0.001, ****p < 0.0001.

Having shown that Th2 cells express high levels of the immunoproteasome (**Figure 1B**), we investigated the potential of targeting the immunoproteasome in a Th2 mediated disease. We used the OVA-AI model and administered ONX 0914 subcutaneously during the challenge phase to analyze the effect of immunoproteasome inhibition on the maturation of Th2 cells (**Figure 3A**). Immunoblot analysis of tissue lysates from spleen, lung and mediastinal lymph nodes revealed a strong LMP7 expression in these organs, which could successfully be inhibited, as indicated by the altered electrophoretic mobility due to the covalent modification with ONX 0914 (22) (**Figure 3B**). Furthermore, it was previously reported that ONX 0914 co-inhibits LMP2 and LMP7 which is required to block autoimmunity (22). We could confirm the inhibition of LMP2 in our setting (**Supplementary Figure S1**). Expression of β5c is rather low in the lung and lymph node, strengthening the importance of targeting LMP7. Of note, the presence of a broader band of β5c in ONX 0914 treated samples, might hint at a partial inhibition of β5c in this setting. Previous studies showed that ONX 0914 binds with a higher specificity to LMP7 than β5c and that in mice at the concentration used, LMP7 is more efficiently inhibited than β5c (10). Nevertheless, from this immunoblot (**Figure 3B**), a partial inhibition of β5c cannot be ruled out and activity assays would be required to ultimately determine the degree of inhibition.

We identified Th2 cells as GATA-3^hi^ or GATA-3^hi^ co-expressing the IL-33 receptor ST2 (35) (**Figure 3C**) which were strongly induced in the lung of OVA-AI mice. We observed an increase of approx. 7-fold compared to naïve mice. ONX 0914 treatment significantly reduced Th2 cells by about 40% whereas the frequency of total CD4^+^ T cells was not altered (**Figures 3D, E** **)**. In line with this, IL-4^+^ CD4^+^ T cells were induced in OVA-AI from about 1.3% in naïve mice to more than 6% in OVA-AI mice treated with the vehicle and the frequency and absolute number of IL-4 expressing CD4^+^ T cells was reduced by 70% in ONX 0914 treated mice. The IL-13^+^ CD4^+^ population was increased from 1.8% in naïve to 2.8% in OVA-AI mice (**Figures 3F-I**). These lower levels of IL-13 compared to IL-4 may result from the fact that IL-4 is expressed earlier and implicated to be important during the early allergic response, whereas IL-13 was described to be involved in the later phases (36). Since the reduction upon ONX 0914 treatment did not reach significance, it will be interesting in the future to see if IL-13 levels may be affected at a later time point. We further restimulated cells from the lymph node and spleen of OVA-AI mice *in vitro* with OVA for four days and observed a reduction of IL-4^+^ T cells by about 50% (**Figure 3J**), indicating a reduced allergen-specific Th2 response in these mice. Viability analysis on day 4 showed that all groups had a viability of about 20% and it was not altered by ONX 0914 treatment (**Supplementary Figure S2**). Since production of IgG1 and IgE antibodies is regulated by Th2 cells (37) and immunoproteasome inhibition reduces activation and antibody secretion of B cells (8, 38–40), we measured anti-OVA IgG1 and IgE serum levels in OVA-AI mice. Compared to naïve mice, we found a strong induction of anti-OVA IgG1 in OVA-AI mice, which was reduced by a third in ONX 0914 treated mice (**Figure 3K**). Levels of anti-OVA IgE were comparably low since class switching to IgE requires more cell divisions then switching to IgG1 (41). The samples showed a high variance, thus statistically significant differences were difficult to detect (**Figure 3L**). Taken together, immunoproteasome inhibition reduced the allergen-specific Th2 response in OVA-AI mice.

### Reduced Infiltration of Inflammatory Cells Upon Immunoproteasome Inhibition

**Figure 4:**
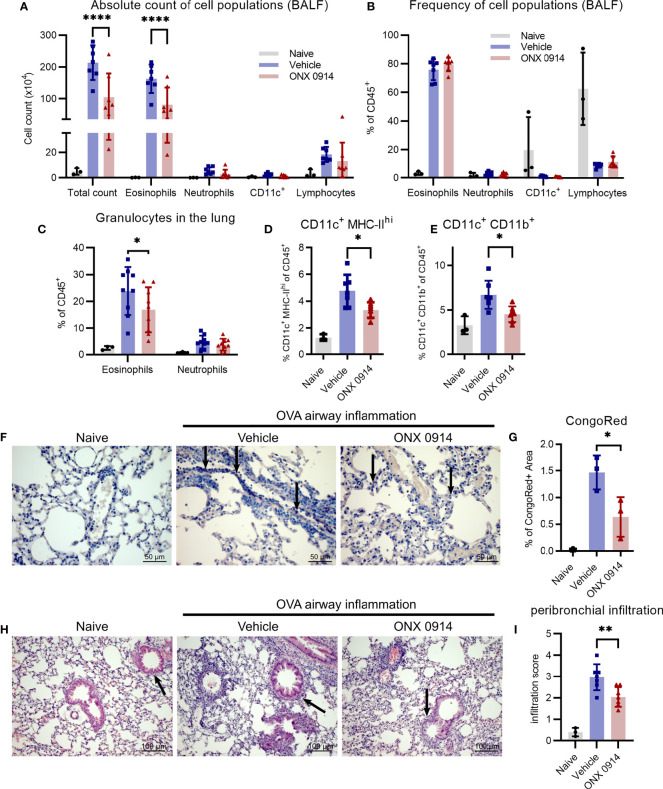
Infiltration of inflammatory cells is reduced upon ONX 0914 treatment in OVA-induced airway inflammation. GATIR mice were sensitized with OVA/Alum by two intraperitoneal (i.p.) injections on day 0 and 7. On day 14, 15 and 16 they were challenged with aerosolized OVA for 20 min. Mice received subcutaneous injections of 10 mg/kg ONX 0914 or vehicle on day 12, 14, 15 and 16 and were analyzed on d17. Naïve mice served as controls. **(A, B)** Infiltrating cells in the broncho-alveolar lavage fluid (BALF) were analyzed by flow cytometry. **(A)** Absolute cell count and **(B)** relative frequency of the different cell populations identified as eosinophils (CD45^+^ CD11c^-^ CD11b^+^ Ly6G^-^ Siglec-F^+^), neutrophils (CD45^+^ CD11c^-^ CD11b^+^ Ly6G^+^), CD11c^+^ and lymphocytes (CD45^+^ CD11b^-^ SSC-A^low^) (naïve: n=3; vehicle/ONX 0914 n=7). Single cell suspensions from lungs were analyzed by flow cytometry and cells were identified as **(C)** eosinophils (CD45^+^ CD11c^-^ CD11b^+^ Ly6G^-^ Siglec-F^+^) and neutrophils (CD45^+^ CD11c^-^ CD11b^+^ Ly6G^+^) (naïve: n=3; vehicle/ONX 0914 n=9) as well as **(D, E)** different CD11c^+^ subpopulations (naïve: n=3; vehicle/ONX 0914 n=7). **(F, G)** Formalin-fixed sections were stained with CongoRed and the positive area was quantified by color deconvolution. Arrows indicate CongoRed-positive cells, representing eosinophils. Scale bar indicates the distance of 50 µm. Data is shown as a representative of two independent experiments with similar results (n=3). **(H, I)** Hematoxilin-eosin-staining of formalin-fixed samples, scale bar indicates the distance of 100 µm (naïve: n=3; vehicle/ONX 0914 n=7). All data is shown as mean ± SD and was pooled from 2-3 independent experiments (except **G**). Significance of differences was analyzed by two-way **(A-C)** or one-way **(D-I)** ANOVA with Holm-Sidak test for multiple comparison. *p <0.05, **p < 0.01, and ****p < 0.0001. For better visibility, only the results for the comparison Vehicle vs. ONX 0914 are indicated.

A hallmark of type 2 airway inflammation is eosinophilic lung infiltration which is mediated by type 2 cytokines like IL-4 and IL-13 (42). To investigate the effect of the Th2 reduction observed in immunoproteasome inhibited mice (**Figures 3D-I**) on the subsequent recruitment of inflammatory cells to the lung, we analyzed the bronchoalveolar lavage fluid (BALF) and lung of OVA-AI mice. Treatment with ONX 0914 reduced the number of infiltrating cells in the BALF by 50%. Flow cytometric analysis shows that the composition of the BALF changed upon OVA-AI induction leading to strong eosinophilic infiltration and ONX 0914 could efficiently reduce the total amount of eosinophils by 50% (**Figure 4A** and **Supplementary Figure S3**) without affecting the relative composition of cell types in the BALF (**Figure 4B**). To confirm induction of allergic inflammation, we included a PBS control group which was sensitized and challenged with PBS without OVA and a challenge control group which was not i.p. sensitized with OVA/Alum but only challenged with OVA aerosol. Both groups showed similar cell counts as naïve mice and infiltration was strongly enhanced in the OVA-AI groups (**Supplementary Figure S4**), indicating that the observed inflammation was antigen-mediated and dependent on previous priming of T cells. Since immune cell populations from the spleen of naïve mice are barely affected by immunoproteasome inhibitors (11, 23), a control group of mice immunized with PBS and treated with ONX 0914 was not included. Similar to the BALF, the frequency of eosinophils in the lung (**Figure 4C** **, -20%**) as well as the proportion of CongoRed-positive area in histologic analyses, indicating eosinophilic infiltration, is reduced upon ONX 0914 treatment (**Figures 4F**, **G, S5C** **; -60%**). In contrast, we did not observe significant changes in the general lymphocyte population in the BALF (**Figure 4A**). Analysis of the GATA-3-vYFP signal allows further separation of these lymphocytes into GATA-3^+^ and GATA-3^-^ (**Supplementary Figure S3**). The GATA-3^+^ lymphocytes are likely mostly T cells which generally express low levels of GATA-3. In contrast, B cells lack GATA-3 expression and therefore probably mainly compose the GATA-3^-^ population (26, 43, 44). We found that more than 90% of lymphocytes in the BALF are GATA-3^+^ and these were not changed upon ONX 0914 treatment as seen for CD4^+^ T cells in the lung (**Figure 3E**). This suggests that in this setting, immunoproteasome inhibition specifically affects Th2 cells rather than lymphocytes in general. Contrary to the BALF, GATA-3^-^ lymphocytes represent about 20% of all CD45^+^ cells in the lung. The relative abundancy of this population did not change with OVA-AI induction and ONX 0914 treatment (**Supplementary Figure S3C**).

The immunoproteasome plays an important role in antigen-presentation (6) and several professional antigen-presenting cells (APCs) are involved in the development of allergic airway inflammation such as dendritic cells (DCs) (45, 46) and macrophages (47). Therefore, we analyzed two different APC subsets characterized by their expression of CD11c, CD11b and major histocompatibility complex class II (MHC-II). The CD11c^+^ MHC-II^hi^ population was strongly induced in OVA-AI mice and ONX 0914 treatment reduced them by a third (**Figure 4D**). Similarly, ONX 0914 limited the increase of the CD11c^+^ CD11b^+^ population seen in OVA-AI mice (**Figure 4E**).

To determine the severity and location of infiltration seen in flow cytometry, lung cross sections were histologically analyzed. In line with the reduction of APCs (**Figures 4D, E** **)**, infiltration of immune cells into peribronchial regions, the location of DC-T cell-interaction (48), was reduced in ONX 0914 treated mice, whereas perivascular infiltration was similar in both groups (**Figures 4H, I** **and** **Supplementary Figure S5A**). Given that type 2 cytokines and macrophages mediate goblet cell hyperplasia, a hallmark for allergic asthma, we analyzed lung sections with PAS-staining and found increased mucus secretion in OVA-AI mice which was only slightly reduced upon ONX 0914 treatment (**Figure S5B**). In summary, we could show that ONX 0914 treatment mitigates inflammatory infiltration into the lung resulting in reduced eosinophilia, not affecting goblet cell hyperplasia.

Since Volkov *et al.* previously reported a similar reduction of inflammation in LMP7-knock out (KO) mice (25), we wanted to confirm this data using exactly the same strain of LMP7 knock-out mice and our similar model of asthma induction. Surprisingly, we were not able to detect any differences between wildtype and LMP7-KO mice regarding BALF and lung infiltration, induction of IL-4^+^ and IL-13^+^ T cells or antibody concentrations (**Supplementary Figure S6**).

### ONX 0914 Reduces the Th2 Response in House Dust Mite-Induced Airway Inflammation

**Figure 5:**
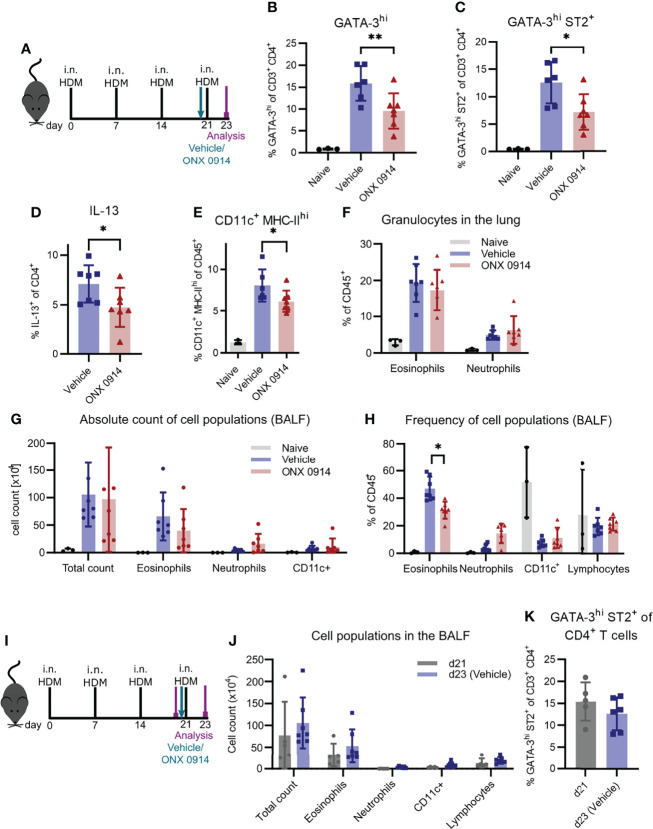
ONX 0914 reduces the Th2 response in house dust mite-induced airway inflammation.** (A-H*)*** GATIR mice received intranasal applications of 50 µg house dust mite (HDM) extract on day 0, 7, 14 and 21. One hour before the last immunization, mice were treated with 10 mg/kg ONX 0914 or vehicle subcutaneously. Inflammatory infiltration was analyzed by flow cytometry on day 23. Naïve mice served as controls. **(A)** Schematic setup of experiments **(B-H)**. Frequency of GATA-3^hi^ **(B)**, GATA-3^hi^ ST2^+^ **(C)** and IL-13^+^ T cells **(D)** in the lung. Frequencies of **(E)** CD11c^+^ MHC-II^hi^ cells as well as **(F)** granulocytes in the lung. **(G)** Absolute cell counts and **(H)** relative frequencies of cell populations in the BALF, identified as eosinophils (CD45^+^ CD11c^-^ CD11b^+^ Ly6G^-^ Siglec-F^-^), neutrophils (CD45^+^ CD11c^-^ CD11b^+^ Ly6G^+^; left), CD11c^+^ and lymphocytes (CD45^+^ CD11b^-^ SSC-A^low^). **(I-K)** Mice of day 23 (d23 (Vehicle)) were immunized and treated as described above. For analysis of the inflammation on day 21 (d21), mice received only three immunizations on day 0, 7 and 14 and were analyzed without treatment and the last immunization. **(I)** Experimental setup for experiments **(J, K)**. **(J)** Infiltration of myeloid cells in the BALF and **(K)** frequency of GATA-3^hi^ ST2^+^ Th2 cells in the lung on day 21 and 23. Data is shown as mean ± SD (naïve: n=3, vehicle/ONX 0914: n=6-7, d21: n=5). *p <0.05 and **p < 0.01. For better visibility, only the results for the comparison vehicle vs. ONX 0914 are indicated. Groups d21 and d23 were not significantly changed.

To substantiate our findings in another, widely used model of airway inflammation, we next analyzed the effect of immunoproteasome inhibition in mice sensitized to HDM, which mimics the disease in humans (**Figure 5A**) (49). To directly compare our study to the previously described results using LMP7-deficient mice, we used the same experimental setup (25). After four intranasal immunizations of GATIR mice, we observed a strong induction of GATA-3^hi^ and GATA-3^hi^ ST2^+^ Th2 cells as well as IL-13^+^ CD4^+^ T cells in the lung (**Figures 5B-D**). Application of a single dose of ONX 0914 one hour before the last immunization strongly reduced the frequency of these cells without affecting the total CD4^+^ T cell frequency. Interestingly, CD11c^+^ MHC-II^hi^ DCs were also reduced by ONX 0914 treatment (**Figure 5E**). In contrast, infiltration of granulocytes in the BALF and lung was barely affected by immunoproteasome inhibition (**Figures 5F-H**). ONX 0914 treatment slightly reduced the frequency of eosinophils in the BALF (**Figure 5H**) but the difference in the absolute cell count was not significantly changed (**Figure 5G**). Of note, there was a tendency of increased neutrophil frequency as well as absolute cell count but this did not reach statistical significance. Since intranasally applied HDM induces local pulmonary inflammation starting from the beginning, we analyzed inflammation at the time point when we usually started treatment (day 21) to evaluate the degree of inflammation before the last immunization and the treatment (**Figure 5I**). We compared this to the inflammation previously seen on day 23 after four immunizations (vehicle group) and found already strong inflammatory infiltration in the BALF on day 21 which was only slightly increased after four immunizations (**Figure 5J** **and** **Supplementary Figure S7**), indicating that ONX 0914 could not have prevented this infiltration by reducing the Th2 response. Interestingly, the frequency of Th2 cells was also already very high on day 21 (**Figure 5K**). The fact that ONX 0914 treatment reduced this frequency on day 23, suggests a specific effect on Th2 cells rather than a general effect on the inflammatory response.

### ONX 0914 Does Not Affect Type 2 Innate Lymphoid Cells in Acute Airway Inflammation

Type 2 innate lymphoid cells (ILC2s) are an innate type of effector lymphocytes which do not express antigen receptors and which are implicated to be early responders in allergic airway inflammation (50, 51). Because ILC2s share many characteristics with Th2 cells and to our knowledge there is no study on the immunoproteasome in ILC2s, we analyzed the effect of immunoproteasome inhibition on these cells in the OVA- and HDM-induced AI models. As reported before, GATIR mice facilitate the identification of ILC2s (52, 53) which also depend on and exhibit high levels of GATA-3 expression (54–56). Although ILC2s could easily be detected (**Supplementary Figure S8A**), we did not observe any differences between the two treatment groups (**Supplementary Figure S8B**), suggesting that these cells do not critically depend on immunoproteasome function.

### Reduced Proportion of Activated but Not Proliferating CD4^+^ T Cells in ONX 0914 Treated Mice

**Figure 6:**
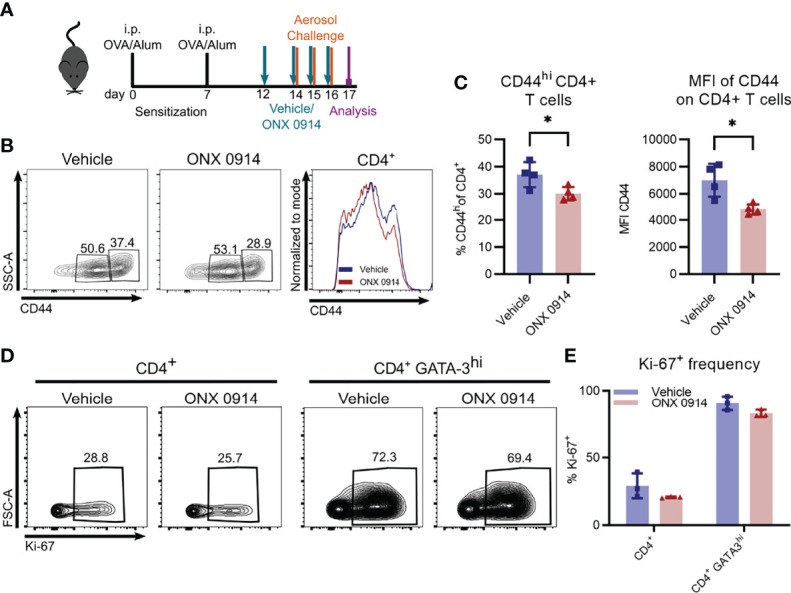
ONX 0914 treatment reduces T cell activation but does not affect the frequency of proliferating cells. GATIR mice were sensitized with OVA/Alum by two intraperitoneal (i.p.) injections on day 0 and 7. On day 14, 15 and 16 they were challenged with aerosolized OVA for 20 min. Mice received subcutaneous injections of 10 mg/kg ONX 0914 or vehicle on day 12, 14, 15 and 16 and were analyzed on day 17. **(A)** Experimental setup. CD44 was analyzed on CD4^+^ T cells in the spleen. Gating **(B)** and frequency **(C)** of CD44^+^ populations of CD4^+^ T cells in the spleen as well as mean fluorescence intensity (MFI) of CD44^+^ on CD4^+^ T cells (right) (n=4). **(D)** Gating of Ki-67 positive populations among all CD4^+^ T cells (left) and CD4^+^ GATA-3^hi^ cells (right) in the lung. **(E)** Quantification of the respective frequency (n=3). Data is shown as mean ± SD as a representative from 2 independent experiments. Significance of differences was analyzed by two-tailed t test **(C)** and two-way ANOVA **(E)**. *p < 0.05.

Since activation of CD4^+^ T cells was previously reported to be affected by inhibition or genetic inactivation of LMP7 (8, 57), we assessed the expression level of CD44 on CD4^+^ T cells in the spleen of OVA-AI mice. CD44 is a cell surface glycoprotein that is upregulated upon antigen-contact and expressed on activated effector and memory T cells (58). We found a reduction of CD44^hi^ cells by ca. 20% in ONX 0914 treated mice (**Figures 6B, C** **)** and a 30% reduction of the expression level of CD44 on CD4^+^ T cells, suggesting a reduced activation of these cells.

Given the reduction of Th2 cells in ONX 0914 treated mice, we investigated the possibility of ONX 0914 affecting their proliferation. Whereas only about 30% of all CD4^+^ T cells express the proliferation marker Ki-67 in the lung, the frequency increased to almost 80% in the GATA-3^hi^ population (**Figures 6D, E** **)**, highlighting the strong proliferative activity of Th2 cells. However, we found no significant differences between the two treatment groups. In addition, *in vitro* restimulation of splenocytes with plate bound anti-CD3 demonstrated equal proliferation capacity of CD4^+^ T cells derived from OVA-AI mice treated with vehicle and ONX 0914 (**Supplementary Figure S9**).

In summary, we could show that immunoproteasome inhibition during the challenge phase reduces the type 2 immune response in mouse models of allergen-induced AI by reducing the activation and cytokine response of CD4^+^ T cells, resulting in a decreased inflammatory eosinophilic airway infiltration in OVA-AI mice.

## Discussion

Several preclinical studies demonstrated the importance of the immunoproteasome in T cell maturation, activation and function as well as effectiveness of immunoproteasome inhibition in T cell mediated (auto-)inflammatory diseases (59, 60). Furthermore, the LMP7/2-selective inhibitor KZR-616 is currently investigated in phase 2 clinical trials for inflammatory diseases (61). Despite extensive research on Th1 and Th17 mediated diseases, only little is known about the role of the immunoproteasome in Th2 cells and previous results vary depending on the way of Th2 induction. *In vitro* polarized Th2 cells were not affected by genetic deletion (25) or pharmacologic inhibition (9) of LMP7. However, a reduced Th2 response was reported in LMP7-KO mice in OVA- but not HDM-induced AI (25) and ONX 0914 did not change the frequency of Th2 cells in the brain in a mouse model for ischemic stroke (14).

Since allergic asthma is a Th2-mediated disease that affects millions of people worldwide (1) and new treatment options are urgently needed for treatment-refractory patients, we aimed at analyzing the therapeutic effect of immunoproteasome inhibition in allergic airway inflammation.

First, we analyzed the immunoproteasome content in Th2 cells as a potential target and found, similar to bulk T cells (8), strong expression of immunoproteasome subunits in different Th subsets. Next, we studied the effect of immunoproteasome inhibition in two different allergic AI mouse models. To this end, we used the novel GATA-3 reporter strain GATIR (26) to identify Th2 cells non-invasively and showed for the first time that the GATA-3-vYFP signal correlates with production of Th2 but not Th1 cytokines or FoxP3 expression not only *in vitro* (26), but also *in vivo*. We found that immunoproteasome inhibition by ONX 0914 during the challenge/effector phase could decrease the Th2 response in OVA-AI. The reduction of Th2 cells was accompanied by decreased IL-4 secretion, IgG1 concentrations as well as inflammatory, mainly eosinophilic, infiltration in the BALF and lung. These observations are in line with earlier reports of short-term standard-/immunoproteasome inhibition by PS-519 (62) or bortezomib (63) which reduced eosinophilic infiltration in OVA-AI models, indicating the relevance of the proteasome in this setting. However, chronic treatment with proteasome inhibitors targeting standard proteasomes and immunoproteasomes alike is too toxic for the therapy of asthma. Selective immunoproteasome inhibition, in contrast, has much reduced adverse side effects (64) due to immunoproteasome expression mainly in immune cells, thus rendering immunoproteasome inhibition an attractive treatment option.

In contrast to the previously reported reduced inflammation in LMP7-KO mice, no difference between wildtype mice and LMP7-KO mice was observed in our setting. Due to increased β5c expression in lungs of LMP7-KO mice (data not shown), we hypothesize that the standard proteasome subunit β5c can substitute LMP7 in Th2 cells in genetically deficient mice as it was reported for naïve and virus-infected LMP7-KO mice (8, 16, 65). Furthermore, recent data has shown that inhibition of both, LMP7 and LMP2, is required to block Th17 function and autoimmunity (22), suggesting a similar mechanism in Th2 cells and a potential explanation for the differences seen between ONX 0914 treatment and LMP7-KO mice. Future studies using subunit specific inhibitors could shed light on this discrepancy between genetic ablation and inhibition. This could further provide insight into the function of the immunoproteasome in T cells as many inhibitors targeting only one subunit were not effective in models of auto-immune diseases (66). Furthermore, the ONX 0914 derivative KZR-616 is already used in clinical studies for systemic lupus erythematosus and could thus be a potential drug to be investigated also in this allergic setting (61).

Furthermore, we saw the reduction of Th2 cells upon immunoproteasome inhibition also in the HDM model in which allergens from HDM trigger an allergic reaction similar to the human disease. Interestingly, the high frequency of Th2 cells on day 21 was markedly reduced after a single dose of ONX 0914 and analysis two days later. One explanation could be killing of Th2 cells by ONX 0914. Since we did not see an effect on the bulk CD4^+^ T cell population and previous data argues against apoptosis induction in CD4^+^ T cells by ONX 0914 (8, 11, 67, 68), this would rather be a Th2 specific effect. However, we were not able to detect apoptosis induction in Th2 cells after ONX 0914 treatment (data not shown). Our results rather suggest that immunoproteasome inhibition reduces the activation of CD4^+^ T cells, as seen by reduced CD44 expression and decreased IL-4^+^ T cells after *in vitro* restimulation, which is in line with previous studies. It has been demonstrated that ONX 0914 reduces the activation of CD4^+^ T cells by restraining ERK signaling and inducing proteostatic stress *in vitro* (8) and that CD4^+^ T cell activation was reduced upon ONX 0914 treatment in a mouse model of myocarditis (57), suggesting a similar mechanism in our setting. Another explanation could be impaired recruitment of T cells to the lung. However, analysis of Th2 cells in the spleen and lymph node did not reveal an accumulation of Th2 cells in both groups (data not shown). Whether reduced activation is the reason for the reduced Th2 response in HDM mice or whether there is another underlying mechanism directly affecting Th2 cells, remains to be elucidated in the future.

Besides a direct influence of ONX 0914 on Th2 cells, an indirect effect through altered activation of CD4^+^ T cells by professional APCs such as DCs could be an underlying mechanism for the reduced Th2 activation. Indeed, we saw a reduction of CD11c^+^ MHC-II^hi^ and CD11b^+^ CD11c^+^ APCs upon ONX 0914 treatment in both AI models. Even though DC activation is impaired by immunoproteasome inhibition and genetic deficiency (69, 70) and immunoproteasome inhibition in pDCs could block proliferation of CD4^+^ T cells *in vitro* (68), airway inflammation developed independently of LMP7 expression in DCs (25). Thus, antigen-specific interaction of DCs and Th2 cells upon immunoproteasome inhibition will be an interesting field for future research.

In contrast to the strong effect of ONX 0914 on Th2 cells, we found only a minor reduction of granulocytes in the BALF and lung of HDM-mice which were already persistent at the time of ONX 0914 treatment. Thus, we hypothesize that granulocytes do not require functional immunoproteasome like it was described in long-term bortezomib treatment of chronic asthma (63). Additionally, the inflammation could be maintained partly by ILC2s (50) which were not affected by ONX 0914 treatment. Even though the effect was rather weak, we could detect a small reduction in the frequency of eosinophils and an increase of neutrophils. Since HDM extracts contain various allergens which trigger allergic responses *via* different pathways leading to eosinophilic and/or neutrophilic infiltration (71–74), it will be interesting in the future to further decipher in which of these reactions the immunoproteasome is involved.

Taken together, we show that immunoproteasome inhibition by ONX 0914 can reduce the Th2 response in models of OVA- and HDM-AI. Our data indicate that Th2 cells are critically dependent on immunoproteasome function and that the impaired Th2 response can further limit the recruitment of inflammatory cells to the lung in the OVA-AI model, highlighting the immunoproteasome as a novel target in allergic airway inflammation. Since a single dose of ONX 0914 was not sufficient to reduce persisting eosinophilic infiltration in the HDM-AI but strongly affected Th2 cells, it will be interesting to study prolonged ONX 0914 treatment in the future to see if limiting the Th2 response reduces the recruitment of eosinophils also in this setting. Further investigations concerning the treatment schedule will be required to fully address this question, e.g. repeated dosing and variation of the starting point. Furthermore, a very wide variety of HDM asthma models, differing not only in timing but also in dosing and the composition of the HDM extract, is described in the literature. Analysis of different HDM models could shed light on the underlying mechanism how and at which time the immunoproteasome is required for the allergic response and the recruitment of inflammatory cells. Nevertheless, our data provides insight into the important role of the immunoproteasome in Th2 cells in allergic airway inflammation and lays the ground for therapeutically targeting the immunoproteasome in this context. Furthermore, KZR-616, an immunoproteasome inhibitor similar to ONX 0914 (61), is currently in clinical trials for treatment of autoimmunity and thus, the effect of immunoproteasome inhibition in allergic asthma can be rapidly assessed in patients.

## Data Availability Statement

The raw data supporting the conclusions of this article will be made available by the authors, without undue reservation.

## Ethics Statement

The animal study was reviewed and approved by Regierungspräsidium Freiburg.

## Author Contributions

FO designed and performed all experiments, analyzed the data and wrote the manuscript. MB provided experimental help and advice, refined the manuscript and supported the submission process. TNR and HJF generated and contributed the GATIR mice before publication. MG conceived experiments, supervised the project and acquired resources. All authors contributed to the refinement of the manuscript and approved the final manuscript prior to submission.

## Funding

This study was funded by the German Research Foundation (DFG) Collaborative Research Center SFB969 project C01. HJF was funded by a grant from the Deutsche Forschungsgemeinschaft (FE 578/3-2) and institutional resources.

## Conflict of Interest

The authors declare that the research was conducted in the absence of any commercial or financial relationships that could be construed as a potential conflict of interest.

## Publisher’s Note

All claims expressed in this article are solely those of the authors and do not necessarily represent those of their affiliated organizations, or those of the publisher, the editors and the reviewers. Any product that may be evaluated in this article, or claim that may be made by its manufacturer, is not guaranteed or endorsed by the publisher.

## Abbreviations

AI, airway inflammation; APC, antigen-presenting cell; BALF, bronchoalveolar lavage fluid; DC, dendritic cell; HDM, house dust mite; ILC2, type 2 innate lymphoid cell; LMP, low molecular weight polypeptide; MECL-1, multicatalytic endopeptidase complex-like; OVA, ovalbumin; vYFP, venus yellow fluorescent protein
