# Supplementary material for: Immunoproteasome Inhibition Reduces the T Helper 2 Response in Mouse Models of Allergic Airway Inflammation
Source: Front Immunol. 2022 May 30;13:870720. doi: 10.3389/fimmu.2022.870720 (PMC9197384; doi:10.3389/fimmu.2022.870720)
Supplement: Supplementary file 1 [file DataSheet_1.pdf]

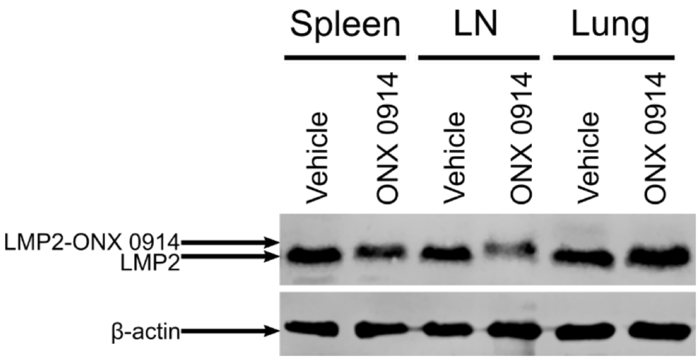

Figure S1 ONX 0914 inhibits the immunoproteasome subunit LMP2. GATIR mice were sensitized with OVA/Alum by two intraperitoneal (i.p.) injections on day 0 and 7. On day 14, 15 and 16 they were challenged with aerosolized OVA for 20 min. Mice received subcutaneous injections of 10 mg/kg ONX 0914 or vehicle on day 12, 14, 15 and 16 and were analyzed on day 17. Lysates of spleen, lymph nodes and lung were analyzed by immunoblotting against the indicated proteins. The shift of electrophoretic mobility of LMP2 results from covalent modification with ONX 0914. β-actin served as a loading control.

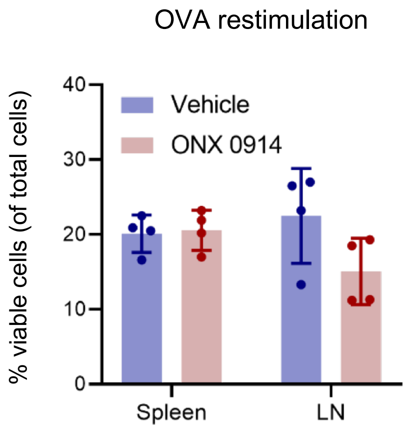

Figure S2 Immunoproteasome inhibition does not affect the viability of *in vitro* restimulated T cells. GATIR mice were sensitized with OVA/Alum by two intraperitoneal (i.p.) injections on day 0 and 7. On day 14, 15 and 16 they were challenged with aerosolized OVA for 20 min. Mice received subcutaneous injections of 10 mg/kg ONX 0914 or vehicle on day 12, 14, 15 and 16 and were analyzed on day 17. Cells from spleen and lymph nodes were restimulated with ovalbumin *in vitro* and analyzed on day 4. Analysis of viable cells by flow cytometry shows no difference between the treatment groups in the total frequency of viable cells. Data is shown as mean ± SD (n=4). Two-way ANOVA with Holm-Sidak post hoc test was performed to determine statistical differences (no significant differences detected).

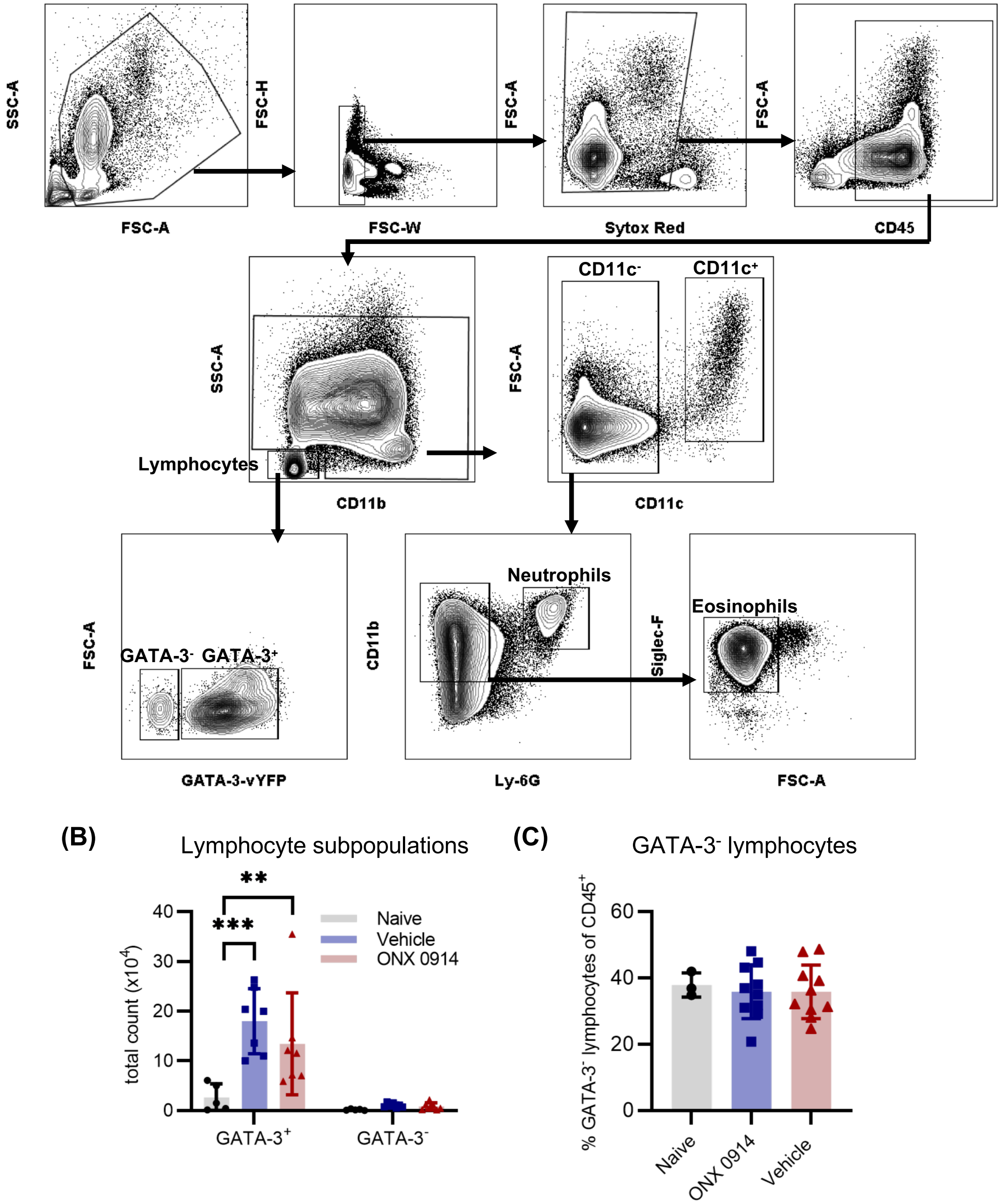

Figure S3 Infiltrating cell populations in the BALF and lung of OVA-AI mice. GATIR mice were sensitized with OVA/Alum by two intraperitoneal (i.p.) injections on day 0 and 7. On day 14, 15 and 16 they were challenged with aerosolized OVA for 20 min. Mice received subcutaneous injections of 10 mg/kg ONX 0914 or vehicle on day 12, 14, 15 and 16 and were analyzed on d17. Naïve mice served as controls. **(A)** Gating scheme for the analysis of infiltrating cells in the BALF (sample from the OVA-AI vehicle group). After the exclusion of debris, doublets and dead cells, cells were gated on $CD45^{+}$. Lymphocytes were identified as $CD11b^{-}$ $SSC\text{-}A^{low}$ and further analyzed for their expression of GATA-3-vYFP. Granulocytes were identified as $CD11c^{-}$, $CD11b^{+}$ $Ly6G^{-}$ (eosinophils) or $CD11b^{+}$ $Ly6G^{+}$ (neutrophils). **(B)** Absolute count of lymphocyte subpopulations in the BALF (naïve: n=5; vehicle/ONX 0914: n=7). **(C)** Relative frequency of $GATA\text{-}3^{-}$ lymphocytes in the lung (naïve: n=3; vehicle/ONX 0914: n=10). Data is shown as mean ± SD. Two-way **(B)** or one-way **(C)** ANOVA with Holm-Sidak post hoc test was performed to determine statistical differences. ** $p < 0.01$, *** $p < 0.001$.

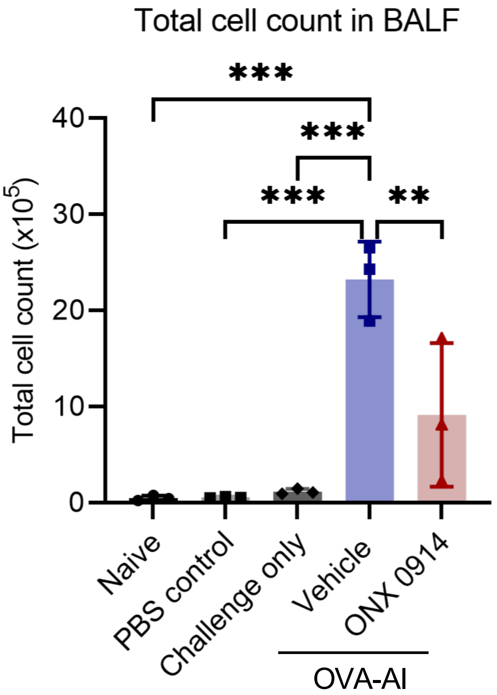

Figure S4 Infiltration of inflammatory cells into the BALF in allergic airway inflammation. For OVA-induced airway inflammation (OVA-AI), GATIR mice were sensitized with OVA/Alum by two intraperitoneal (i.p.) injections on day 0 and 7. On day 14, 15 and 16 they were challenged with aerosolized OVA for 20 min. Mice received subcutaneous injections of 10 mg/kg ONX 0914 or vehicle on day 12, 14, 15 and 16. PBS control mice received i.p. injections of PBS/alum on day 0 and 7 and were challenged with PBS only for three times on day 14-16; they did not receive vehicle or ONX 0914 treatment. All mice were analyzed on d17. "Challenge only" mice were not sensitized but only subjected three times to OVA aerosol and analyzed 1 day after the third challenge. Naïve mice were left untreated. Data is shown as mean ± SD (n=3). One-way ANOVA with Holm-Sidak post hoc test was performed to determine statistical differences, ** $p < 0.01$, *** $p < 0.001$.

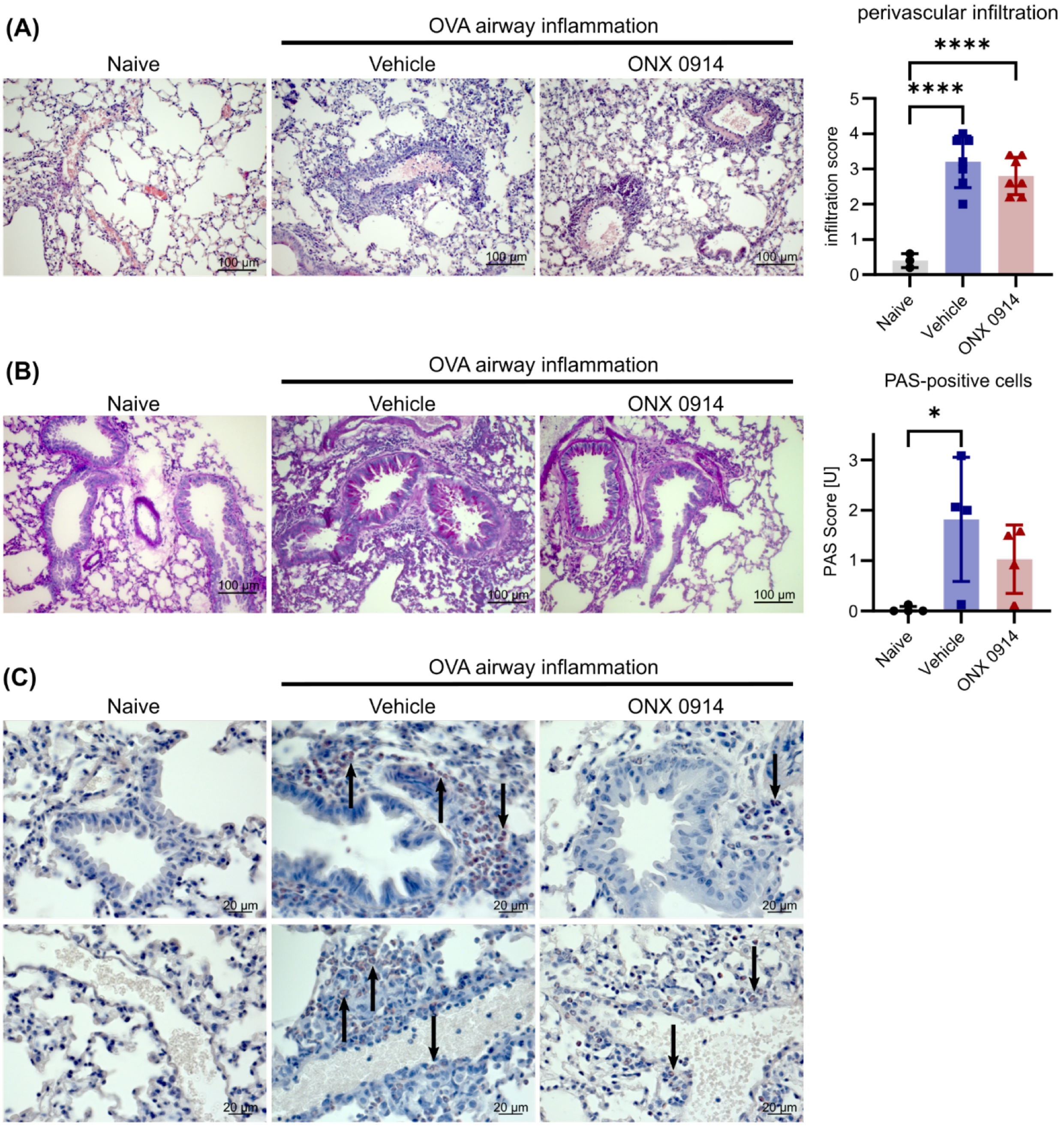

Figure S5 Infiltration of inflammatory cells and goblet hyperplasia upon ONX 0914 treatment in OVA-induced airway inflammation. GATIR mice were sensitized with OVA/Alum by two intraperitoneal (i.p.) injections on day 0 and 7. On day 14, 15 and 16 they were challenged with aerosolized OVA for 20 min. Mice received subcutaneous injections of 10 mg/kg ONX 0914 or vehicle on day 12, 14, 15 and 16 and were analyzed on d17. Naïve mice served as controls. **(A)** Hematoxilin-eosin-staining (naïve: n=3; vehicle/ONX 0914: n=7) and **(B)** PAS-staining of formalin-fixed lung samples (naïve: n=3; vehicle/ONX 0914: n=4) with representative micrographs and scoring results. Scale bar indicates the distance of 100 µm. **(C)** High magnification of CongoRed stained lung samples. Upper row shows peribronchial regions, lower row perivascular ones. Arrows indicate CongoRed-positive cells, representing eosinophils. Scale bar indicates the distance of 20 µm. Data is shown as mean ± SD. * $p < 0.05$ and ****$p < 0.0001$.

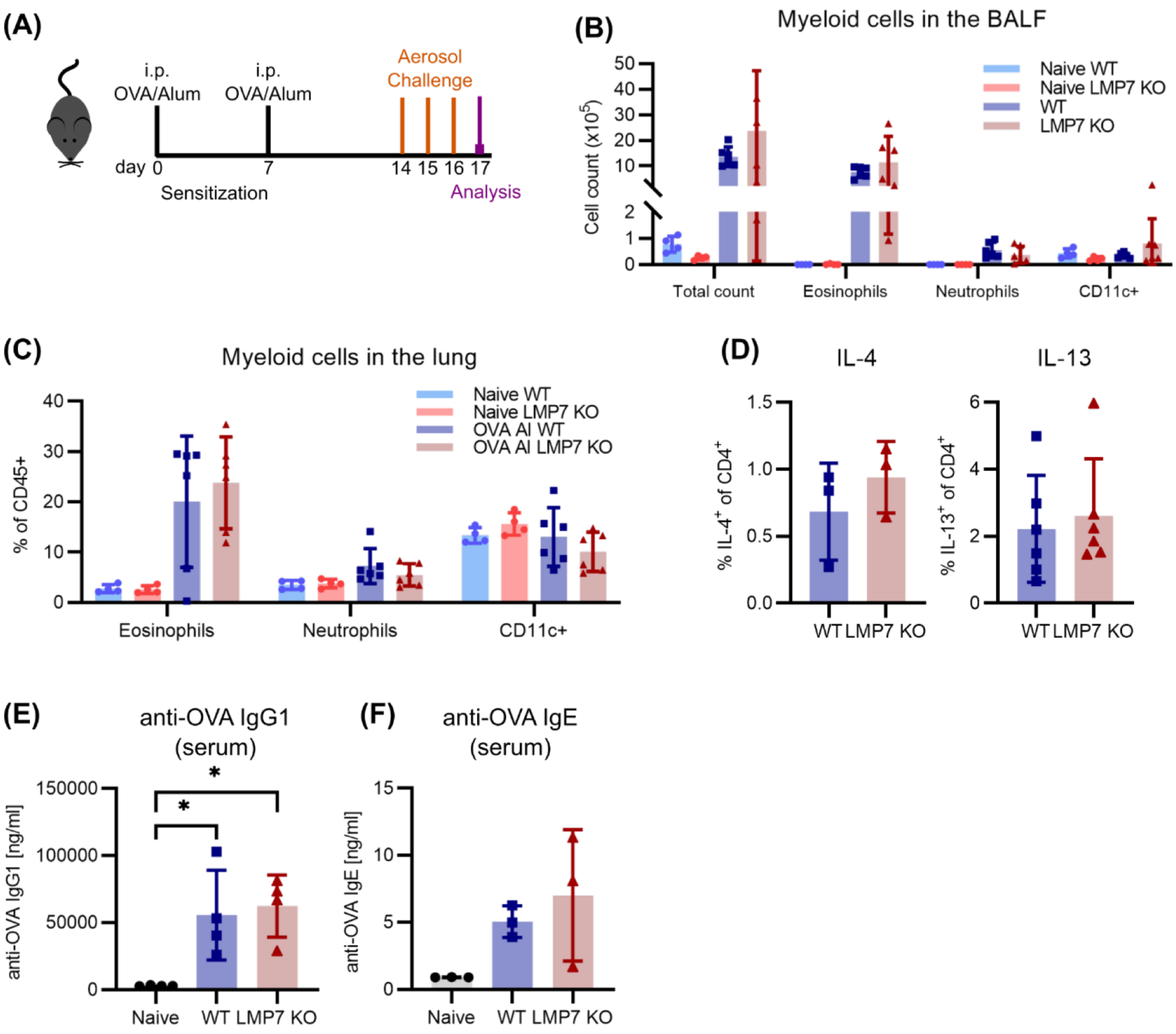

Figure S6 LMP7-deficiency does not impair the type 2 response in ovalbumin induced airway inflammation. Wildtype (WT) and LMP7-knock-out (KO) mice were sensitized with OVA/Alum by two intraperitoneal (i.p.) injections on day 0 and 7. On day 14, 15 and 16 they were challenged with aerosolized OVA for 20 min. Mice were analyzed on day 17 and naïve mice served as controls. **(A)** Experimental setup. **(B)** Single cell suspensions from lungs and **(C)** the broncho-alveolar lavage fluid (BALF) were analyzed by flow cytometry and cells were identified as eosinophils ($CD45^+$ $CD11c^-$ $CD11b^+$ $Ly6G^-$ Siglec-F^+), neutrophils ($CD45^+$ $CD11c^-$ $CD11b^+$ $Ly6G^+$) and $CD11c^+$. **(D)** Frequency of IL-4 and IL-13 expressing $CD4^+$ cells in the lung. Titers of anti-OVA IgG1 **(E)** and IgE **(F)** in the serum. Data is shown as mean ± SD, pooled from two independent experiments (naïve: n=3-4; OVA-AI: n=3-6). Significance of differences was analyzed by **(B, C)** two-way or **(E, F)** one-way ANOVA with Holm-Sidak post hoc test. **(D)** was analyzed by two-tailed t test. * $p < 0.05$.

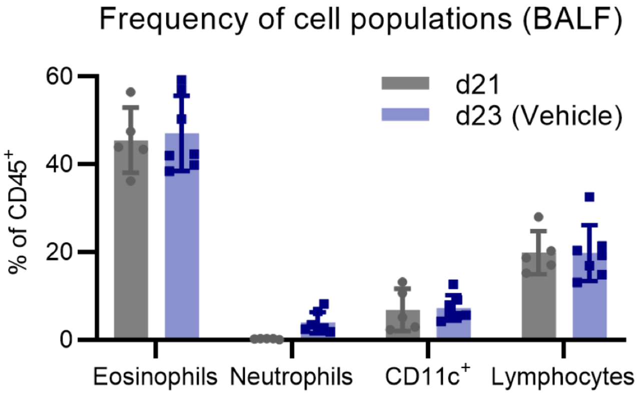

Figure S7 GATIR mice of group d23 (vehicle) received intranasal applications of 50 µg house dust mite (HDM) extract on day 0, 7, 14 and 21. One hour before the last immunization, mice were treated with vehicle subcutaneously and inflammatory infiltration was analyzed by flow cytometry on day 23. For analysis of the inflammation on day 21 (d21), mice received only three immunizations on day 0, 7 and 14 and were analyzed without treatment and the last immunization on day 21. Frequency of cell populations in the BALF, identified as eosinophils ($CD45^+$ $CD11c^-$ $CD11b^+$ $Ly6G^-$ Siglec-F^-), neutrophils ($CD45^+$ $CD11c^-$ $CD11b^+$ $Ly6G^+$; left), $CD11c^+$ and lymphocytes ($CD45^+$ $CD11b^-$ SSC-A^{low}). Data is shown as mean ± SD (d21: n=5; d23 (vehicle): n=7). There were no significant differences detected between the groups (two-way ANOVA with Holm-Sidak post hoc test).

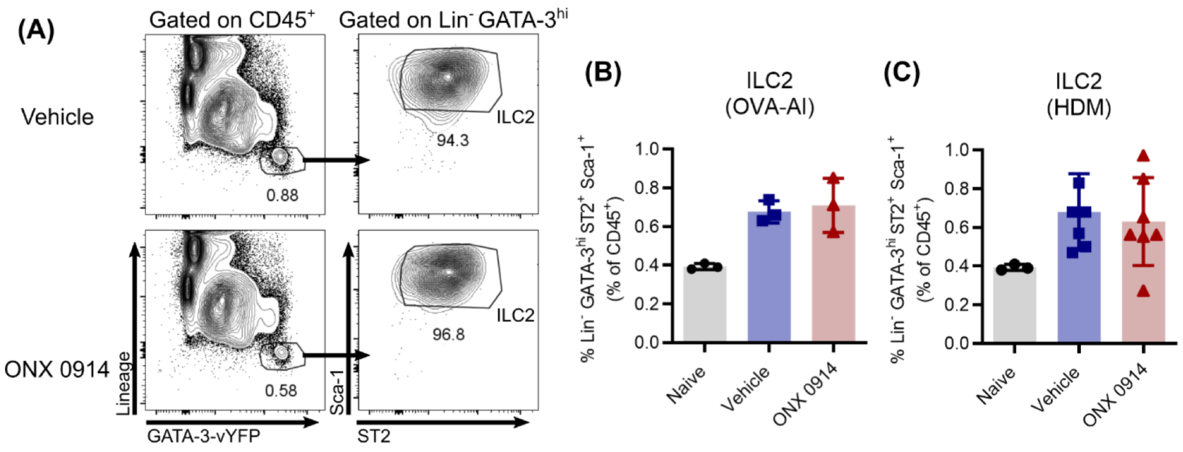

Figure S8 ONX 0914 does not affect type 2 innate lymphoid cells in acute airway inflammation. Allergic airway inflammation was induced in GATIR mice using ovalbumin (OVA; A,B) and house dust mite extract (HDM; C). Type 2 Innate lymphoid cells (ILC2s) were identified as $CD45^+$ $Lineage^-$ GATA-3-$vYFP^{hi}$ Sca-1^+ $ST2^+$. Data is shown as mean ± SD, pooled from one **(B)** or two **(C)** independent experiments (naïve: n=3; vehicle/ONX 0914: **(B)** n=3 and **(C)** n=7).

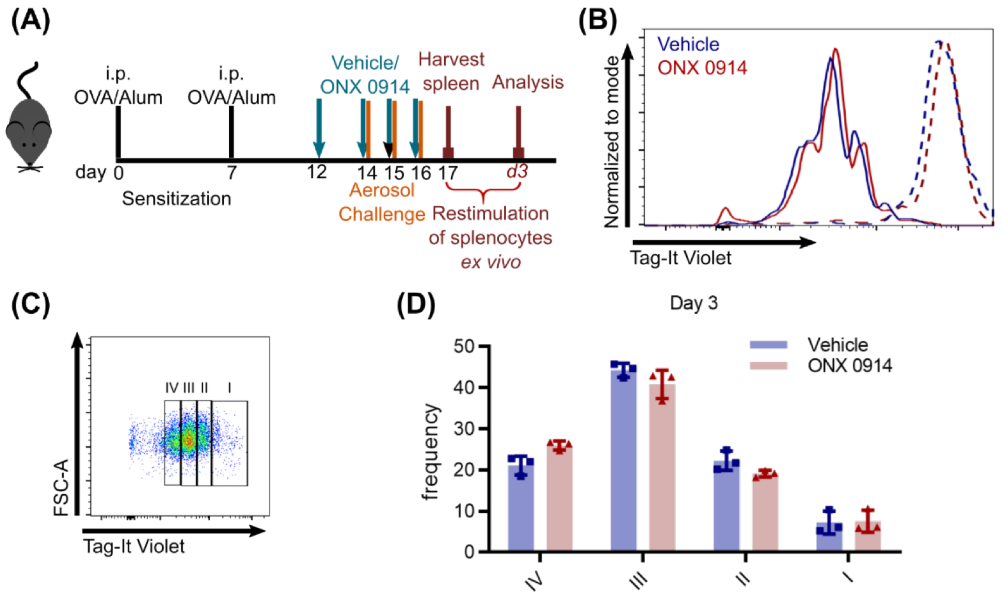

Figure S9 Proliferation of CD4$^+$ T cells is not affected by ONX 0914 treatment. GATIR mice were sensitized with OVA/Alum by two intraperitoneal (i.p.) injections on day 0 and 7. On day 14, 15 and 16 they were challenged with aerosolized OVA for 20 min. Mice received subcutaneous injections of 10 mg/kg ONX 0914 or vehicle on day 12, 14, 15 and 16. Spleens were harvested on day 17, stained with Tag-It Violet cell trace dye and restimulated *ex vivo* with anti-CD3 for 3 days. **(A)** Experimental setup. **(B)** Representative fluorescence signals of splenocytes on day 0 (dashed line) and day 3 (solid line) from vehicle (blue) and ONX 0914 treated (red) mice. **(C)** Gating strategy for the identification of populations with different amounts of cell divisions and **(D)** quantification of the populations. Data is shown as mean ± SD, n=3.

Table **S1**: List of antibodies used.

**Antigen**	**Fluorochrome**	**Clone**	**Supplier**
CD3	APC	145-2C11	Biolegend
CD3	eFluor450	145-2C11	eBioscience
CD4	BV605	GK1.5	Biolegend
CD4	PE	GK1.5	Biolegend
CD4	APC	GK1.5	Biolegend
CD11b	BV605	M1/70	Biolegend
CD11c	PE-Cy7	N418	eBioscience
CD44	PE-Cy7	IM7	eBioscience
CD45	eFluor450	30-F11	eBioscience
CD45	BV605	30-F11	Biolegend
CD62L	APC	MEL-14	Biolegend
ST2	PE-Cy7	DIH4	Biolegend
Siglec-F	PerCP-Cy5.5	E50-2440	BD Biosciences
Ly6G	AlexaFluor700	1A8	BD Biosciences
MHC-II	BV421	M5/114.15.2	Biolegend
Lineage Cocktail	BV421	17A2, RB6-8C5, RA3-6B2, Ter-119, M1/70	Biolegend
Sca-1	PE	REA422	Miltenyi Biotech
Ki-67	eFluor660	SolA15	eBioscience
FoxP3	eFluor450	FJK-16s	eBioscience
CD3	unlabeled	145-2C11	Biolegend
CD28	unlabeled	37.51	Biolegend
IL-4	PE	11B11	Biolegend
IL-13	PE	eBio13A	invitrogen
IL-17A	APC	ebio17B7	eBiosciences
IFN-γ	BV421	XMG1.2	Biolegend
β1c		E1K90	Cell Signaling
β2c		E1L5H	Cell Signaling
β5c		D1H68	Cell Signaling
LMP7		polyclonal	Khan S, van den Broek M, Schwarz K, de Giuli R, Diener P-A, Groettrup M. Immunoproteasomes Largely Replace Constitutive Proteasomes During an Antiviral and Antibacterial Immune Response in the Liver. J Immunol. 2001;167(12):6859 - 6868. doi:10.4049/jimmunol.167.12.6859
LMP2		polyclonal	abcam
MECL-1		polyclonal	Basler M, Guillaume B, Kolb C, et al. Reduced Immunoproteasome Formation and Accumulation of Immunoproteasomal Precursors in the Brains of Lymphocytic Choriomeningitis Virus-Infected Mice. J Immunol. 2010, 1001517; doi:10.4049/jimmunol.1001517
γ-tubulin		GTU-88	Sigma-Aldrich
β-actin		polyclonal	Sigma-Aldrich
